# Association between composite dietary antioxidant index and metabolic dysfunction associated steatotic liver disease: result from NHANES, 2017-2020

**DOI:** 10.3389/fnut.2024.1412516

**Published:** 2024-07-22

**Authors:** Zhaofu Zhang, Hao Wang, Youpeng Chen

**Affiliations:** Department of Infectious Diseases, The Seventh Affiliated Hospital, Sun Yat-sen University, Shenzhen, China

**Keywords:** metabolic dysfunction associated steatotic liver disease, composite dietary antioxidant index, oxidative stress, liver stiffness, restricted cubic spline

## Abstract

**Background:**

The development of metabolic dysfunction associated steatotic liver disease (MASLD) has been associated with lipid accumulation, oxidative stress, endoplasmic reticulum stress, and lipotoxicity. The Composite Dietary Antioxidant Index (CDAI) is a comprehensive score representing an individual intake of various dietary antioxidants, including vitamin A, vitamin C, vitamin E, selenium, zinc, and carotenoids. This study investigated the association between CDAI and MASLD.

**Materials and methods:**

Clinical and demographic data, as well as ultrasound transient elastography measurements at baseline, were collected from the National Health and Nutrition Examination Survey 2017–2020 (NHANES 2017–2020). The controlled attenuation parameter was utilized to diagnose the presence of hepatic steatosis and to categorize individuals into those with and without MASLD. Liver stiffness was measured by ultrasound transient elastography, and subjects were classified as those with and without advanced liver fibrosis.

**Results:**

This study included 5,884 adults, of whom 3,433 were diagnosed with MASLD, resulting in a weighted prevalence of 57.3%. After adjusting for covariates, the odds ratios for MASLD were 0.96 (95% CI: 0.82, 1.12) in the second quartile, 0.80 (95% CI: 0.68, 0.95) in the third quartile and 0.60 (95% CI: 0.49, 0.73) in the fourth quartile, respectively. CDAI, however, was not significantly associated with advanced liver fibrosis.

**Conclusion:**

These findings suggested that scores on the CDAI were linearly and negatively associated with the prevalence of MASLD in the United States adults.

## 1 Introduction

In June 2023, non-alcoholic fatty liver disease (NAFLD) and metabolic associated fatty liver disease (MAFLD) were renamed metabolic dysfunction associated steatotic liver disease (MASLD) to emphasize the role of metabolic dysfunction in its development (1). MASLD has been estimated to affect 30% of the worldwide adult population. Metabolic dysfunction associated steatohepatitis (MASH), a step within the broad spectrum of lesions included in MAFLD, is histologically defined by the presence of lobular inflammation and hepatocellular ballooning, and associated with an increased risk of progression to fibrosis (2). Recently, the Food and Drug Administration (FDA) in the United States approved the drug Resmetirom for the treatment of MASH in adult patients, the first medication approved by the FDA for MASH in 40 years. Lifestyle adjustments are also important in the treatment of MASH (3, 4).

Currently, lifestyle interventions to achieve weight loss remain the cornerstone of MASLD management (5–13). Oxidative stress has been shown to play a significant role in the development of MASLD (14). A case–control study in Iran, enrolling 158 NAFLD patients and 357 healthy controls, revealed that the prevalence of NAFLD was significantly lower in subjects within the highest tertile than in subjects within the lowest tertile of dietary total antioxidant capacity (DTAC), after adjusting for potential confounding factors (odds ratio [OR] 0.19; 95% confidence interval [CI] 0.9, 0.34; *p* for trend <0.001) (15). DTAC, however, can itself be influenced by various factors, including seasonality, geographic location, storage conditions, and access to water and sunlight. Additionally, food preparation and cooking methods can also impact DTAC. These considerations were addressed by the formulation of the Composite Dietary Antioxidant Index (CDAI), a reliable and valid measurement of the overall antioxidant composition of an individual’s diet. The CDAI is a comprehensive score of multiple dietary antioxidants, including vitamins A, C, and E, selenium, zinc, and carotenoids, representing an individual’s overall dietary intake of antioxidants (16). A number of studies have demonstrated a negative association between CDAI and the prevalence of diabetes, hypertension, chronic kidney disease, and depression (17–20), as well as all-cause and cardiovascular mortality in the general United States population (21).

Although dietary antioxidants have been shown effective in the treatment of adverse health effects, including oxidative stress (22–26), the specific relationship between the CDAI and MASLD remains unclear. Based on the hypothesis that the CDAI is negatively associated with MASLD, the present study investigated the potential correlation between CDAI and MASLD using data from the National Health and Nutrition Examination Survey (NHANES) 2017–2020.

## 2 Methods

### 2.1 Study population and design

**Figure 1:**
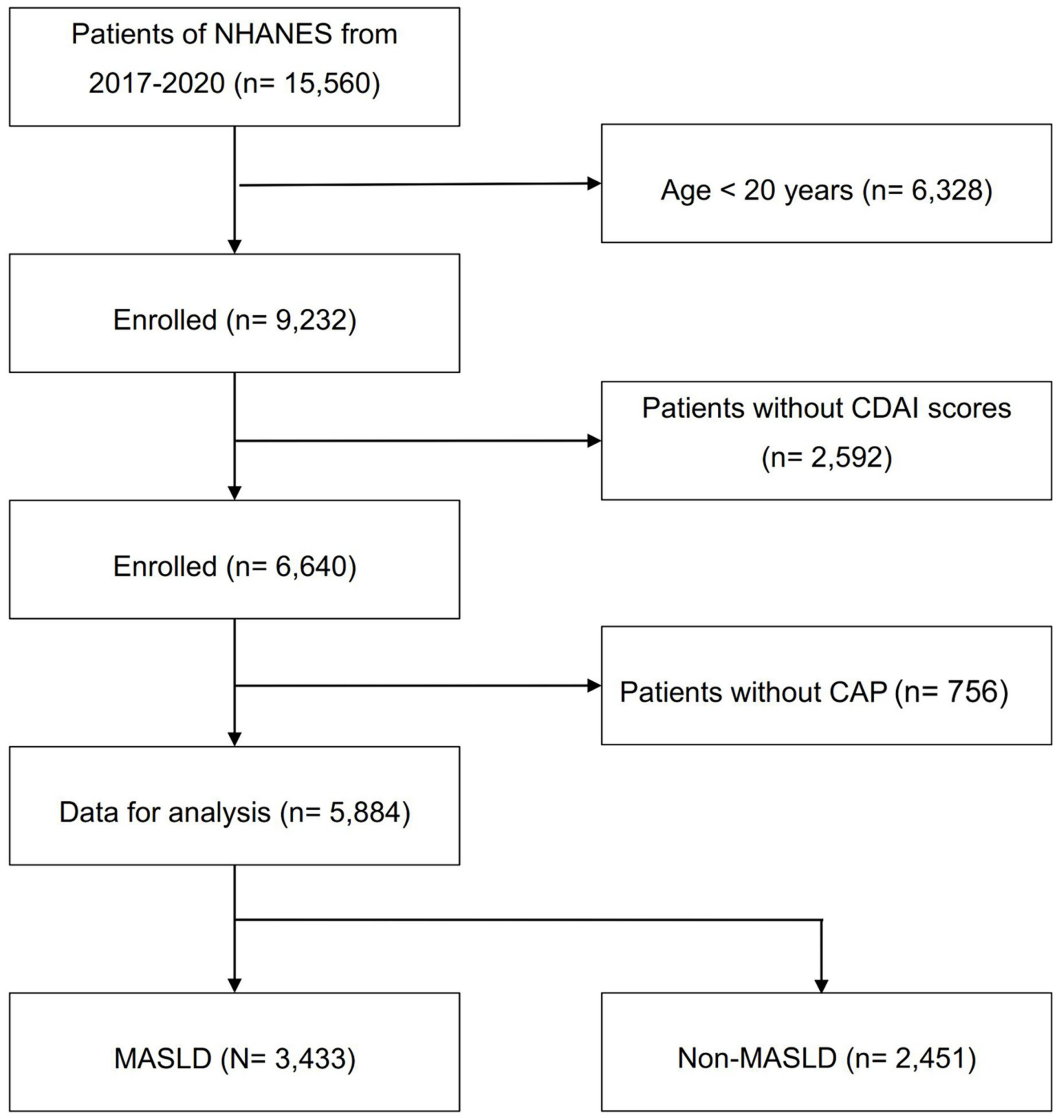
Flowchart of participant selection. NHANES, National Health and Nutrition Examination Survey; CAP, controlled attenuation parameter; CDAI, composite dietary antioxidant index; MASLD, metabolic dysfunction associated steatotic liver disease.

The study population consisted of subjects enrolled in the NHANES 2017–2020, due to the availability of liver ultrasound transient elastography data in this population. Using the Fibroscan to estimate the severity of liver steatosis and fibrosis (27), hepatic steatosis was defined as a controlled attenuation parameter (CAP) ≥ 248 dB/m, with a sensitivity of 68.8% and a specificity of 82.2% (28). Advanced liver fibrosis was defined as a liver stiffness measure (LSM) value ≥8.0 kPa (29). The study ultimately enrolled 5,884 subjects from an initial population of 15,560. Subjects aged <20 years (*n* = 6,328), those with missing CDAI data (*n* = 2,592), and those with missing Fibroscan measurements (*n* = 756) were excluded. According to the latest consensus diagnostic criteria from the EASL-AASLD-ALEH consensus statement in 2023 (1), 3,433 people were diagnosed with MASLD, while the others were included in the health control group (*n* = 2,451). The details are presented in Figure 1. The study protocol was approved and documented by the Research Ethics Review Committee of the National Center for Health Statistics of the United States. Before participants were enrolled in the study, they had to provide written informed consent.

### 2.2 Definition of CDAI

Date were obtained from participants in the NHANES database, through two 24-h dietary recalls conducted at separate time points (30). The first recall was taken in-person at a mobile testing center, while the second recall was performed 3–10 days later via a telephone consultation. The CDAI of each participant was calculated based on the dietary recall of intake of six dietary antioxidants, vitamin A, vitamin C, vitamin E, zinc, selenium, and carotenoids (31).

### 2.3 Assessment of covariates

Referring to a previous study (32), the covariates included age, sex, race, education level, marital status, income status, physical activity, smoking status, alcohol consumption, and energy intake. Because other variables, such as blood glucose concentration, blood pressure, lipid profile, and body mass index were considered in the diagnosis of MASLD, these variables were not included in the regression model. Marital status was divided into married and unmarried; and education level into less than high school, high school, and more than high school. Income status was assessed by calculating the household income to poverty income ratio (PIR), with subjects divided into those with low (< 1.0), moderate (1.0–3.0), and high (≥ 3.0) PIR. Smoking status was categorized as never, former or current; physical activity was categorized as never, moderate and vigorous exercise (33); heavy drinking was defined as more than 14 drinks/week for men or more than 7 drinks/week for women (34). Detailed explanations of the methods used to calculate these variables are available on the NHANES website.^1^

### 2.4 Statistical analysis

All analyses considered the weights of each variable in the NHANES database. Continuous variables were described as weighted mean (standard error) and categorical variables were described as weighted n (weighted percentage). The CDAI was converted into quartiles, the relationship between CDAI and MASLD/advanced liver fibrosis was examined by three multivariate logistic regression models, all of which using the first CDAI quartile group as reference. Model 1 was a crude model without any adjustment for covariates; Model 2 included adjustments for age, sex, and race; and Model 3 included the adjustments in Model 2 as well as adjustments for education level, marital status, PIR, smoking, alcohol consumption, physical activity, and energy intake. To further eliminate relevant confounding factors, stratified analyses were performed based on factors such as age, sex, race, PIR, energy intake, and ethnicity. All analyses were performed using R software (version 4.3.2, Vienna, Austria: R Foundation for Statistical Computing, 2016) and Stata/SE software, v.16 (StataCorp, College Station, TX, USA), with bilateral *p* values <0.05 defined as statistically significant.

## 3 Results

### 3.1 Baseline characteristics of the enrolled population

The study included a total of 5,884 eligible participants, out of which 3,433 were diagnosed with MASLD, resulting in a weighted prevalence of 57.3% (92,050,707/160,526,094). Compared to the non-MASLD participants, MASLD participants had higher age and energy intake as 51.5 ± 0.4 years vs. 44.4 ± 0.5 years and 2,112 ± 20.0 kcal/day vs. 2,039 ± 23.9 kcal/day, respectively (*p* < 0.001). The proportion of male (51.4% vs. 43.8%, *p* < 0.001) and of married individuals (67.6% vs. 56.6%, *p* < 0.001) was significantly higher in MASLD. However, the MASLD group had a significantly lower score on CDAI than the non-MASLD group (0.6 ± 0.1 vs. 1.1 ± 0.1, *p* < 0.001). Race, educational level and smoking status also differed significantly in these two groups (all *p* < 0.05) (Table 1).

**Table 1:** Characteristics of the enrolled population.

Characteristics	Non-MASLD (*n* = 2,451)	MASLD (*n* = 3,433)	*p* value
Age (years; SD)	44.4 (0.5)	51.5 (0.4)	<0.001
Male, *n* (%)	29,992,219 (43.8%)	47,314,063 (51.4%)	<0.001
Race/ethnicity, *n* (%)			<0.001
Mexican American	3,834,621 (5.6%)	8,652,766 (9.4%)	
Other Hispanic	5,067,178 (7.4%)	6,627,650 (7.2%)	
Non-Hispanic White	43,824,247 (64.0%)	59,556,807 (64.7%)	
Non-Hispanic Black	9,791,980 (14.3%)	8,928,918 (9.7%)	
Non-Hispanic Asian	3,629,195 (5.3%)	4,234,332 (4.6%)	
Other Race-Including Multi-Racial	2,328,166 (3.4%)	4,050,244 (4.4%)	
Married, *n* (%)	38,757,069 (56.6%)	92,050,700 (67.6%)	<0.001
Educational level, *n* (%)			<0.001
less than high school or high school	21,775,173 (31.8%)	34,519,015 (37.5%)	
more than high school	46,700,214 (68.2%)	57,531,692 (62.5%)	
Poverty-income ratio, *n* (%)			0.08
low	7,189,915 (10.5%)	9,481,222 (10.3%)	
moderate	25,404,368 (37.1%)	37,556,688 (40.8%)	
high	35,881,104 (52.4%)	45,012,797 (48.9%)	
Smoker, *n* (%)			<0.001
Never	41,906,936 (61.2%)	51,916,598 (56.4%)	
Former	14,790,683 (21.6%)	25,498,045 (27.7%)	
Current	11,777,768 (17.2%)	14,636,064 (15.9%)	
Physical activity, *n* (%)			0.80
Never	31,909,530 (46.6%)	42,159,223 (45.8%)	
Moderate	17,940,551 (26.2%)	25,037,792 (27.2%)	
Vigorous	18,625,306 (27.2%)	24,853,692 (27.0%)	
Heavy drinker, *n* (%)	26,773,876 (39.1%)	33,322,355 (36.2%)	0.06
Energy intake (Kcal/day; SD)	2039 (23.9)	2,112 (20.0)	<0.001
CDAI	1.1 (0.1)	0.6 (0.1)	<0.001

Continuous variables are expressed as weighted mean [standard error (SD)], categorical variables as weighted n (weighted percentage). The *p*-values for the continuous variables were calculated by weighted linear regression model, while the categorical variables were calculated by weighted chi-square test.

MASLD, metabolic dysfunction associated steatotic liver disease; CADI, composite dietary antioxidant index.

### 3.2 Association of CDAI with MASLD

Weighted multivariate logistic regression analyses were performed to further access the relationship between CDAI and MASLD based on the quartile of CDAI. Univariate logistic regression analysis showed that the unadjusted OR (95% CI) of MASLD was significantly lower in the fourth quartile (Q4) (OR 0.83; 95% CI: 0.72, 0.96, *p* = 0.013) than in the first quartile (Q1) (used as reference). After adjusting for potential confounding factors, the risk of MASLD in Model 3 remained significantly lower in Q4 than in Q1 (OR 0.60; 95% CI: 0.49, 0.73, *p* < 0.001). In addition, there was a significant negative correlation between the CDAI index and the occurrence of MASLD for all three models (Table 2).

**Table 2:** Association of composite dietary antioxidant index and metabolic dysfunction associated steatotic liver disease.

CDAI	Model 1	Model 2	Model 3
	OR (95% CI), *p*	OR (95% CI), *p*	OR (95% CI), *p*
Q1	1.00 (Reference)	1.00 (Reference)	1.00 (Reference)
Q2	1.12 (0.97, 1.30), 0.12	1.07 (0.92, 1.25), 0.39	0.96 (0.82, 1.12), 0.60
Q3	0.99 (0.86, 1.16), 0.97	0.95 (0.82, 1.11), 0.53	0.80 (0.68, 0.95), 0.012
Q4	0.83 (0.72, 0.96), 0.013	0.79 (0.68, 0.92), 0.003	0.60 (0.49, 0.73), <0.001
*p* for trend	0.004	<0.001	<0.001

Model 1: unadjusted.

Model 2: adjusted for age, gender and race.

Model 3: adjusted for Model 2 plus age, gender, race, marital status, education level, poverty income ratio, alcohol intake, smoking, physical activity, and energy intake.

Metabolic dysfunction associated steatotic liver disease was defined by controlled attenuation parameter ≥ 248 dB/m.

CDAI, composite dietary antioxidant index; Q, quartile; OR, odds ratio; CI, confidence interval.

**Figure 2:**
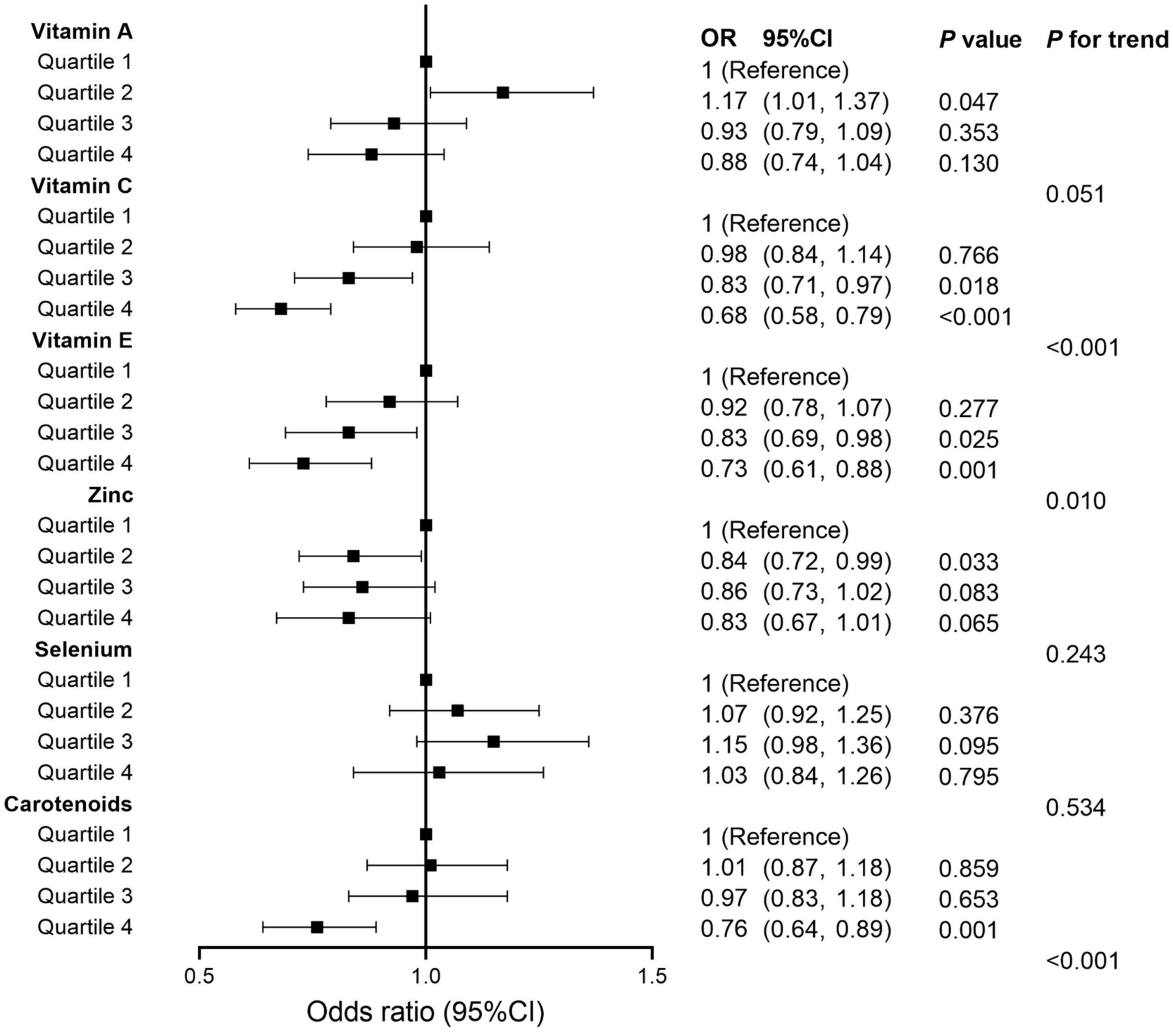
Forest plot for analyzing the relationship between each component of composite dietary antioxidant index and metabolic dysfunction associated steatotic liver disease. OR, odds ratio; CI, confidence interval.

**Figure 3:**
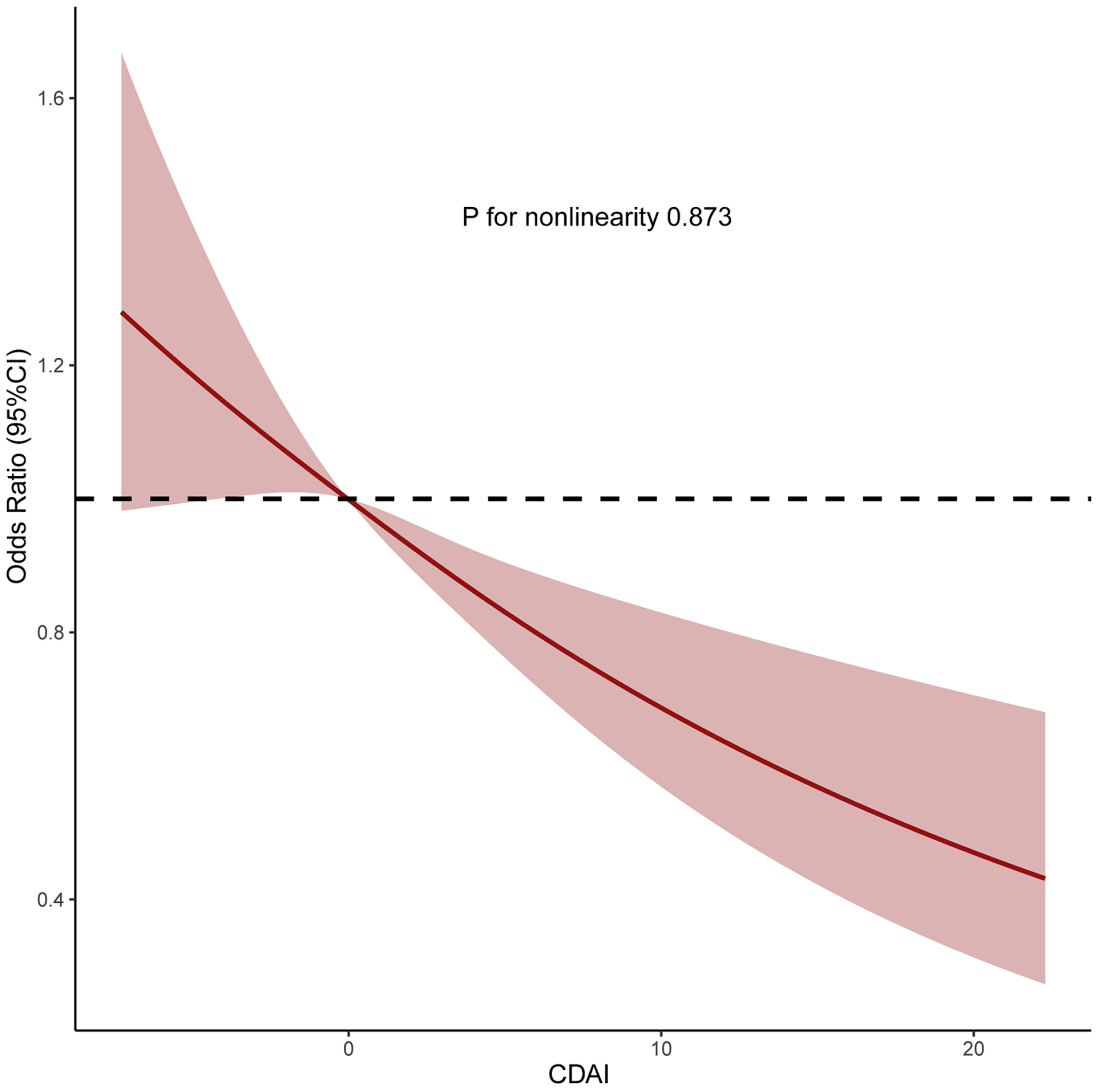
Restricted cubic spine model of the odds ratio of metabolic dysfunction associated steatotic liver disease with composite dietary antioxidant index. All were adjusted for age, sex, race, marital status, education level, poverty income ratio, alcohol intake, smoking, physical activity, and energy intake. CI, confidence interval.

We also analyzed separately the impact of each component of CDAI upon MASLD risk. These analyses found that vitamin C, vitamin E, and carotenoids may play an important role in the negative correlation between CDAI and MASLD (all *p* for trend <0.05) (Figure 2). Subsequently, restricted cubic spline (RCS) analysis to investigate the dose–response relationship between CDAI and MASLD risk showed that higher levels of CDAI were associated with a reduced likelihood of MASLD, with a non-linear *p*-value of 0.873 (Figure 3).

**Figure 4:**
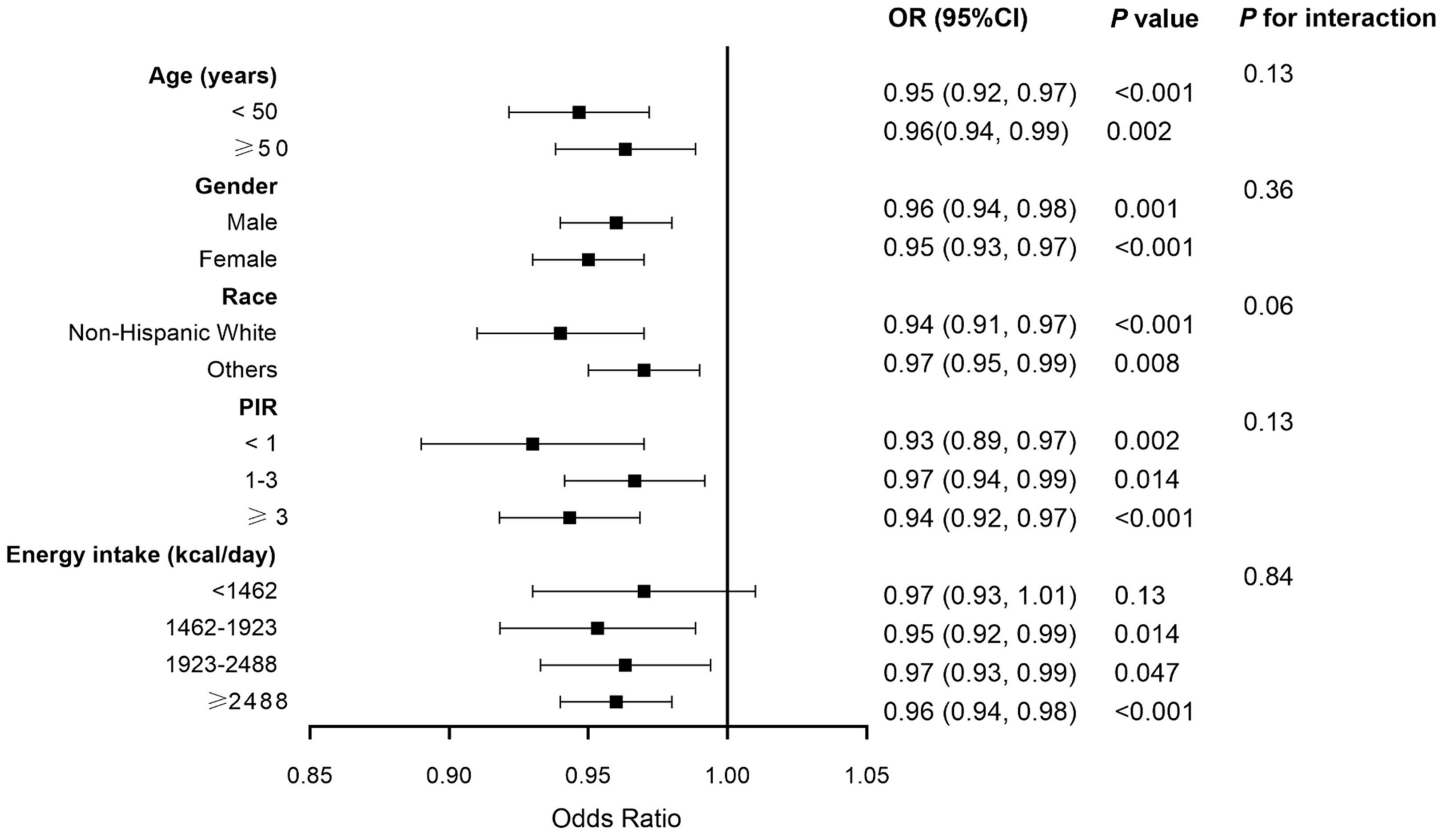
Forest plot of stratified analyses of the relationship between composite dietary antioxidant index and metabolic dysfunction associated steatotic liver disease. Analyses were adjusted for covariates age, gender, race, marital status, education level, poverty income ratio, alcohol intake, smoking, physical activity, and energy intake, when they were not the strata variables. OR, odds ratio; CI, confidence interval.

Sensitivity analyses were used to assess the stability of the relationship between CDAI and MASLD. The fatty liver index (FLI) has been shown relatively effective in detecting MASLD in the United States population (35). The use of an FLI ≥ 60 to diagnose MASLD (36, 37) showed a significant negative correlation between CDAI and MASLD in all three models (Table 3). Even after excluding subjects with extremely high (> 8,000 kcal/day for men, > 5,000 kcal/day for women) and low (< 500 kcal/day for both sexes) daily energy consumption, a significant negative correlation between CDAI and the prevalence of MASLD persisted in all three models (Table 4). To rule out the possibility that higher CDAI may result from the intake of multivitamin and multimineral supplements, vitamin B6, vitamin D, vitamin K, calcium, phosphorus, and magnesium intake were included as covariates. However, CDAI and the prevalence of MASLD still showed a significant negative correlation in all three models (Table 5). Stratified analyses to explore the association between CDAI and MASLD by age, sex, race, PIR, and energy intake (converted to quartiles) showed that none of these variables significantly modified the relationship between CDAI and MASLD (*p* for interaction ≥0.05; Figure 4).

**Table 3:** Association of composite dietary antioxidant index and metabolic dysfunction associated steatotic liver disease defined by fatty liver index.

CDAI	Model 1	Model 2	Model 3
	OR (95% CI), *p*	OR (95% CI), *p*	OR (95% CI), *p*
Q1	1.00 (Reference)	1.00 (Reference)	1.00 (Reference)
Q2	0.93 (0.81, 1.08), 0.068	0.92 (0.79, 1.06), 0.262	0.87 (0.74, 1.02), 0.084
Q3	1.01 (0.88, 1.17), 0.850	1.01 (0.87, 1.16), 0.980	0.98 (0.83, 1.16), 0.830
Q4	0.79 (0.68, 0.91), 0.001	0.78 (0.68, 0.91), 0.001	0.73 (0.59, 0.88), 0.001
*p* for trend	0.007	0.006	0.002

Model 1: unadjusted.

Model 2: adjusted for age, gender and race.

Model 3: adjusted for Model 2 plus age, gender, race, marital status, education level, poverty income ratio, alcohol intake, smoking, physical activity, and energy intake.

Metabolic dysfunction associated steatotic liver disease was defined by fatty liver index ≥ 60.

CDAI, composite dietary antioxidant index; Q, quartile; OR, odds ratio; CI, confidence interval.

**Table 4:** Association of composite dietary antioxidant index and metabolic dysfunction associated steatotic liver disease excluding individuals with extreme energy intake.

CDAI	Model 1	Model 2	Model 3
	OR (95% CI), *p*	OR (95% CI), *p*	OR (95% CI), *p*
Q1	1.00 (Reference)	1.00 (Reference)	1.00 (Reference)
Q2	1.13 (0.97, 1.31), 0.11	1.08 (0.92, 1.25), 0.36	0.96 (0.81, 1.12), 0.57
Q3	0.99 (0.86, 1.16), 0.99	0.96 (0.82, 1.15), 0.57	0.79 (0.67, 0.94), 0.008
Q4	0.84 (0.72, 0.97), 0.017	0.79 (0.68, 0.93), 0.004	0.59 (0.48, 0.72), <0.001
*p* for trend	0.001	<0.001	<0.001

Model 1: unadjusted.

Model 2: adjusted for age, gender and race.

Model 3: adjusted for Model 2 plus age, gender, race, marital status, education level, poverty income ratio, alcohol intake, smoking, physical activity, and energy intake.

Metabolic dysfunction associated steatotic liver disease was defined by controlled attenuation parameter ≥ 248 dB/m.

CDAI, composite dietary antioxidant index; Q, quartile; OR, odds ratio; CI, confidence interval.

**Table 5:** Association of composite dietary antioxidant index and metabolic dysfunction associated steatotic liver disease after multivitamin and multimineral adjustment.

CDAI	Model 1	Model 2	Model 3
	OR (95% CI), *p*	OR (95% CI), *p*	OR (95% CI), *p*
Q1	1.00 (Reference)	1.00 (Reference)	1.00 (Reference)
Q2	1.12 (0.97, 1.30), 0.12	1.07 (0.92, 1.25), 0.39	0.99 (0.84, 1.17), 0.923
Q3	0.99 (0.86, 1.16), 0.97	0.95 (0.82, 1.11), 0.53	0.85 (0.71, 1.02), 0.080
Q4	0.83 (0.72, 0.96), 0.013	0.79 (0.68, 0.92), 0.003	0.69 (0.55, 0.88), 0.002
*p* for trend	0.004	<0.001	<0.001

Model 1: unadjusted.

Model 2: adjusted for age, gender and race.

Model 3: adjusted for Model 2 plus age, gender, race, marital status, education level, poverty income ratio, alcohol intake, smoking, physical activity, energy intake, vitamin B6, vitamin D, vitamin K, calcium, phosphorus and magnesium.

Metabolic dysfunction associated steatotic liver disease was defined by controlled attenuation parameter ≥ 248 dB/m.

CDAI, composite dietary antioxidant index; Q, quartile; OR, odds ratio; CI, confidence interval.

### 3.3 Association of CDAI with advanced liver fibrosis

Weighted multivariate logistic regression analysis showed that, without any adjustment, the prevalence of advanced liver fibrosis was lower in CDAI Q4 (OR 0.99; 95% CI: 0.69, 1.43, *p* = 0.98) than in Q1 (as reference), but this difference was not statistically significant. After adjusting for confounding factors, similarly no statistically significant difference was found between Q4 and Q1 in Model 3 (OR 0.87; 95% CI: 0.54, 1.41, *p* = 0.56). These findings are presented in Table 6.

**Table 6:** Association of composite dietary antioxidant index and advanced liver fibrosis.

CDAI	Model 1	Model 2	Model 3
	OR (95% CI), *p*	OR (95% CI), *p*	OR (95% CI), *p*
Q1	1.00 (Reference)	1.00 (Reference)	1.00 (Reference)
Q2	1.04 (0.72, 1.49), 0.84	0.99 (0.68, 1.43), 0.96	0.97 (0.66, 1.42), 0.86
Q3	0.81 (0.56, 1.18), 0.27	0.77 (0.53, 1.11), 0.16	0.73 (0.48, 1.13), 0.16
Q4	0.99 (0.69, 1.43), 0.98	0.96 (0.67, 1.39), 0.84	0.87 (0.54, 1.41), 0.56
*p* for trend	0.11	0.24	0.07

Model 1: unadjusted.

Model 2: adjusted for age, gender and race.

Model 3: adjusted for Model 2 plus age, gender, race, marital status, education level, poverty income ratio, alcohol intake, smoking, physical activity, and energy intake.

CDAI, composite dietary antioxidant index; Q, quartile; OR, odds ratio; CI, confidence interval.

## 4 Discussion

Increases in the prevalence of obesity and metabolic diseases have been associated with increases in the incidence of MASLD. Lifestyle modifications such as exercise and dietary interventions have been found effective in treating MASLD, but their underlying mechanisms remain unclear. To our knowledge, the present study is the first to explore the relationship between CDAI and MASLD in a large sample population. This study showed a significant negative linear association between CDAI scores and the risk of developing MASLD, even after adjusting for covariates.

MASLD is a complex multi-factorial disease, associated with various genetic, epigenetic, and environmental factors, with its pathogenesis being incompletely understood (38–40). The “multiple hit” hypothesis involving many potentially concurrent factors may provide a suitable explanation of MASLD. Oxidative stress, one of the factors contributing to these hits (39, 41), is a reflection of an imbalance between the generation of reactive species and the clearing capacity of the antioxidant system, favoring the former (42). At high concentrations, reactive species can induce oxidative modifications in cellular macromolecules (DNA, lipids, proteins, etc.), leading to the accumulation of damaged macromolecules and triggering liver injury (43, 44). Oxidative stress assessment involves (1) direct measurement of reactive species levels, (2) measurement of oxidative damage to biomolecules, and/or (3) assessment of antioxidant status. Many antioxidant biomarkers have been applied to evaluate the redox status of MASLD (44).

Pro-inflammatory diets can amplify the inflammatory responses within the body by increasing oxidative stress and immune dysregulation. Unhealthy dietary habits are often associated with higher levels of inflammatory cytokines, which can promote the development of atherosclerosis (45). The CDAI, which measures the dietary intake of antioxidants such as vitamins A, C, and E, selenium, zinc, and carotenoids, has been used to measure dietary antioxidant intake, population norms, and the overall impact of antioxidants on health outcomes (16, 46). Although individual antioxidants may play a role in the pathogenesis of MASLD, biological interactions among dietary antioxidants should also be considered (47–50). In our study, CDAI was measured to estimate combined exposure to six dietary antioxidants, resulting in a potential dose–response association between combined antioxidant intake and MASLD risk. That is, increased CDAI was associated with a lower risk of MASLD.

Several previous studies have assessed the relationship between intake of a specific vitamin with MASLD (51–54). These studies found that vitamin C, vitamin E, and carotenoids were negatively associated with the risk of MASLD, while vitamin A was positively associated with the risk of MASLD (51–54). In our study, a negative correlation between vitamin C, vitamin E, and carotenoids and the risk of developing MASLD was also found. Nevertheless, the results of our study did not indicate a significant correlation between vitamin A and MASLD (although a strong tendency was found *p* = 0.051). Furthermore, the present study found that the CDAI, a widely validated comprehensive antioxidant index, correlated negatively with the risk of developing MASLD.

To our knowledge, our study was the first to assess the relationship between CDAI and MASLD in a large national sample, finding a significant negative correlation between the two. Although the present study did not directly investigate the underlying mechanisms, several possibilities should be considered. Oxidative stress is a key factor in the pathogenesis of MASLD (9, 10, 39, 55). Antioxidant intake can reduce the overall level of oxidative stress (56–58). Using the CDAI index as a comprehensive assessment of dietary antioxidants may be a tool able to more comprehensively assess the relationship between the redox state and the pathogenesis of MASLD.

The present study had several limitations. Its cross-sectional design and lack of information on time of diagnosis prevented the determination of a causal relationship between CDAI and MASLD. Secondly, although the models controlled for covariates, all relevant covariates may not have been considered. Thirdly, due to the nature of the NHANES database, information on dietary intake was self-reported, which may have introduced recall bias. Large-scale prospective studies are therefore needed to better understand the relationship between the CDAI and MASLD.

## 5 Conclusion

This study demonstrated a significant negative association between CDAI scores and MASLD in United States adults. Moreover, these results suggested that diet rich in antioxidants may reduce the risk of MASLD, but additional studies in clinical cohorts are needed to confirm these findings.

## Data availability statement

The datasets presented in this study can be found in online repositories. The names of the repository/repositories and accession number(s) can be found below: the survey data are publicly available on the internet for data users and researchers throughout the world (www.cdc.gov/nchs/nhanes).

## Ethics statement

The studies involving humans were approved by the parts of this study that involved human participants, human materials, or human data were conducted in compliance with the Declaration of Helsinki and were approved by the National Center for Health Statistics (NCHS) Ethics Review Board. The patients/participants provided written informed consent to participate in this study. The studies were conducted in accordance with the local legislation and institutional requirements. The participants provided their written informed consent to participate in this study.

## Author contributions

ZZ: Data curation, Formal analysis, Methodology, Writing – original draft, Writing – review & editing. HW: Visualization, Writing – original draft, Writing – review & editing. YC: Funding acquisition, Project administration, Supervision, Writing – review & editing.

## Footnotes

^1^ https://www.cdc.gov/nchs/nhanes/

## Abbreviations

CAP: Controlled attenuation parameter
CDAI: Composite Dietary Antioxidant Index
CI: Confidence interval
DTAC: Dietary total antioxidant capacity
FDA: Food and Drug Administration
FLI: Fatty liver index
LSM: Liver stiffness measurement
MAFLD: Metabolic associated fatty liver disease
MASH: Metabolic dysfunction associated steatohepatitis
MASLD: Metabolic dysfunction associated steatotic liver disease
NAFLD: Non-alcoholic fatty liver disease
NHANES: National Health and Nutrition Examination Survey
ORs: Odds ratios
PIR: Poverty income ratio
Q: Quartile
RCS: Restricted cubic spline
